# Anticestodal Activity of Endophytic *Pestalotiopsis* sp. on Protoscoleces of Hydatid Cyst *Echinococcus granulosus*

**DOI:** 10.1155/2013/308515

**Published:** 2013-08-25

**Authors:** Vijay C. Verma, Mayank Gangwar, Madhu Yashpal, Gopal Nath

**Affiliations:** ^1^Laboratory of Gastrointestinal Infections and Molecular Diagnosis, Department of Microbiology, Institute of Medical Sciences, Banaras Hindu University, Varanasi, Uttar Pradesh 221 005, India; ^2^Department of Pharmacology, Institute of Medical Sciences, Banaras Hindu University, Varanasi, Uttar Pradesh 221005, India; ^3^Electron Microscope Facility, Department of Anatomy, Institute of Medical Sciences, Banaras Hindu University, Varanasi, Uttar Pradesh 221005, India

## Abstract

Surgery is still the main treatment in hydatidosis caused by *Echinococcus*, which is a global health problem in human and animals. So, there is need for some natural protoscolicidal agents for instillation to prevent their reoccurrence at therapeutic doses. In this present investigation, anticestodal activity of one of the endophytic fungi *Pestalotiopsis* sp. from Neem plant was observed on protoscoleces of hydatid cysts of *Echinococcus granulosus*. Viability of protoscoleces was confirmed by 0.1% aqueous eosin red stain method, where mortality was observed at different concentrations with respect to time. An average anticestodal activity was observed with different endophytic fungal strains, that is, *Nigrospora* (479 ± 2.9), *Colletotrichum *(469 ± 25.8), *Fusarium *(355 ± 14.5), and *Chaetomium* (332 ± 28.3) showing 64 to 70% protoscolicidal activity, except *Pestalotiopsis *sp. (581 ± 15.0), which showed promising scolicidal activity up to 97% mortality just within 30 min of incubation. These species showed significant reduction in viability of protoscoleces. This is the first report on the scolicidal activity of endophytic *Pestalotiopsis* sp. We conclude that ultrastructural changes in protoscoleces were due to endophytic extract suggesting that there may be some bioactive compounds that have selective action on the tegument layer of protoscoleces. As compared with that of standard drug used, endophytic species of Neem plant shows significant anticestodal activity.

## 1. Introduction

Fungal endophytes are rapidly emerging as a vital source of isolation and characterization of bioactive natural products that exhibit wide spectrum of chemical diversity and their associated bioactivities [1–3]. Recent developments in endophytic research provide exciting platform for the isolation and characterization of novel bioactive natural products and activities associated such as anticancer [4, 5] antifungal [6] to provide new prodrugs for major health challenges. Another very interesting aspect of endophyte bioprospection is their potential of producing host mimetic chemicals that are originally known to be produced by the host plant only [7–9]. This potential is quite interesting since many medicinal plants have been overexploited for their therapeutic potential which in turn causes serious concern for their survival. So, this symbiotic relationship offers opportunity to harvest bioactive constituents of medicinal plants from their endophytic symbionts, and by doing so we can not only harvest the bioactive components of rare medicinal plants but also preserve the valuable biodiversity that are about to extinct. We have a long tradition of using medicinal plants to cure many diseases as mentioned in our traditional medical system called “Ayurveda.” Unfortunately, despite recent development in endophyte bioprospection for various kinds of activity like antibacterial, anticancer, anti-inflammatory, and so forth, rare reports are available about the antiparasitic potential of endophytes. More than 2 billion people of tropical and sub-tropical countries are facing serious health problems caused by lymphatic filariasis, onchocerciasis, echinococcosis, and other helminthic infections. Increasing side effects and appearance of resistance to the synthetic anthelmintics stimulates researchers for exploration of novel natural alternatives from medicinal plants, utilizing traditional knowledge base of many Ayurvedic and Unani medical treatment systems.

In India alone, there are hundreds of medicinal plants, and their products are reported to be beneficial in the treatment or control of many parasitic infections for the last few decades, but it could not be developed into viable drugs for a variety of reasons [10]. Thus, it does require urgent steps to find out new natural, safe, and sustainable alternative to the chemical anthelmintics that can be designed as new therapeutic prodrug for parasitic infections. Scanty of reports are available about the potential of endophytes as antiparasitic agent; a few data too that have been reported very recently encourage us to explore the possibilities of new antiparasitic molecules from endophytes [11]. Hydatidosis or cystic echinococcosis (CE) is a chronic zoonosis affecting humans as well as domestic animals [12], caused by the larval stage of a cosmopolitan parasitic cestode *Echinococcus granulosus *[13, 14]. Because of the slow progression of the disease, it might be asymptomatic initially or with very little clinical manifestations [15], which further progresses depending on the site of infection and size of cyst [16]. Despite development in our understanding about the infection of hydatid parasites, we are still facing many challenges for its cure and diagnosis [17]. New drug leads from sources not earlier explored will be one of the aspects to find new alternative to diagnose the hydatid diseases. Although many other medicinal plants are known to have activity against *Echinococcus granulosus *such as *Zataria multiflora *[18]*, Saturena khuzestanica *[19]*, Salvia officinalis, Thymus vulgaris* [20], *Mentha piperita* [21], and *Trachyspermum ammi *[22], but their associated endophytes need attention for the same activity. Thus, due consideration is required for reinvestigation and scientific exploration of medicinal plants for their associated endophytes to find out new anthelmintics alternatives that can overcome the side effects besides being sustainable and environmentally acceptable.

In the present study, we have investigated several endophytic fungal strains isolated from *Azadirachta indica* A. Juss. and came up with a strain of endophytic *Pestalotiopsis* sp. with significant anticestodal activity. This is the first report of its kind that deals with thorough screening of fungal endophytes for anticestodal potential against hydatid cysts *E. granulosus*.

## 2. Materials and Methods

### 2.1. Plant Samples

Random samples of *Azadirachta indica* A. Juss. were collected from across the campus of Banaras Hindu University, Varanasi (25.5°N 82.9°E). Fresh plant samples of healthy and symptomless leaves, stems, and roots were collected. The cut ends were wrapped with parafilm before they were placed in sterile polythene bags, for further isolation procedures in the laboratory.

### 2.2. Surface Treatment, Isolation, and Culture Conditions

A total of 90 tissue segments, 30 each from leaf, stem, and root representing ten different hosts from different ecological settings were sampled. Sequential dipping into 70% ethanol for 1 min, 5% NaOCl for 5 min, and 96% ethanol for 0.5 min, followed by three rinses in sterile distilled water was performed for surface treatment following our previously established method [7, 23, 24]. All sample tissues were then placed onto separate petri dishes (60 mm) containing potato dextrose agar (PDA) supplemented with 250 mg L^−1^ oxytetracycline hydrochloride (Terramycin, Pfizer) for isolating endophytic fungi. All petri dishes were sealed with sterile parafilm to protect them from contamination during repeated handling while examining endophytes and from desiccation. The plates were incubated at 26 ± 2°C and 98% relative humidity (under 12 h fluorescent light/12 h dark light), enclosed in translucent white cover plastic boxes in a BOD cum humidity incubator for 25 days. Fungi that grew from the tissue fragments were subcultured onto fresh PDA plates.

### 2.3. Preliminary Screening of Endophytes for Scolicidal Activity

After isolation of a battery of endophytic microbes from Neem plant, random selections of five endophytic fungi *Nigrospora* sp., *Pestalotiopsis* sp., *Colletotrichum* sp., *Fusarium* sp., and *Chaetomium* sp. were performed for preliminary evaluation of their potential anticestodal activity. The rationale of selecting these fungi is their easy morpho-taxonomic identification at genera level, so that to have clear delineation when working with them and also to avoid molecular identification at initial stage. These strains have very specific and strong morphological features unique to them that make their easy identification from any standard reference manual of morpho-taxonomic identification of fungi [25–28]. These selected strains were cultured in liquid potato dextrose broth (PDB) for 20 days. Filtrate was extracted with equal volume of ethyl acetate twice and dried *in vacuo*. The extracts obtained for each fungus were then dissolved into 0.9% phosphate buffer saline (PBS) at pH 7.3 to make two doses, low 5 mg/mL and high 20 mg/mL, to test anticestodal activity. This preliminary screening suggests significant scolicidal activity with *Pestalotiopsis *sp., thus, we decided to perform further purification of the extract of *Pestalotiopsis* sp. and also perform individual experiments with this strain thereafter.

### 2.4. Partial Purification of *Pestalotiopsis* sp. Extract

The endophytic fungus *Pestalotiopsis *sp. was grown in 2 L of potato dextrose broth (PDB) flasks at 25 ± 2°C for 21 days in BOD cum incubator. The fungal biomass was then removed from flasks by filtration, and the culture fluid was extracted twice with equal volume of ethyl acetate (EtOAc). The EtOAc fraction was dried by flash evaporation, and the yield of crude material was up to 5.7 g. This material was dissolved in a minimal volume of methanol and was subjected to silica gel (size 60–120 mesh, Ranbaxy) column chromatography on a 3 × 30 cm column, with an EtOAc-hexane solvent gradient system. The first 100 mL eluted from the column was discarded, and the next 500 mL eluting from the column (possessing bioactivity) was taken to dryness (3.3 g); a small fraction of this elute was washed with pure hexane several times to remove the oil fraction and placed on a second silica gel column (identical in size to the first column). It was eluted with MeCN/acetone/H_2_O (1 : 1 : 0.5), and 8 subfractions (20 mL) were collected. These subfractions were evaporated under vacuum to obtain dry extracts which were evaluated for their scolicidal potential against protoscoleces of hydatid cysts *E. granulosus*.

### 2.5. Collection of Cysts

Hydatid cysts of *E. granulosus* were collected aseptically from infected liver and lungs of cattles slaughtered in an abattoir located in Varanasi City, India. The intact cysts were immediately placed in an icebox and transported within 3 h to the laboratory at Department of Microbiology, Institute of Medical Sciences, Banaras Hindu University, Varanasi, India. Cysts were washed 3 times in sterile phosphate buffered saline (0.9% PBS), and pH 7.2. Cyst surfaces were sterilized by 70% ethanol and were cut open, and vesicle fluid containing protoscoleces was separated from the metacestode tissue and host adventitia. The fertility of cysts was determined by the presence of free protoscoleces in cystic fluid by microscopic examination of a wet mount drop. Hydatid fluid along with protoscoleces was collected as previously described by Smyth and Barrett [29].

### 2.6. Preparation and Culture of Protoscoleces

Hydatid fluid containing protoscoleces was allowed to settle completely into 15 mL Falcon tubes without centrifugation, and it was left to settle for an hour to obtain hydatid sand at room temperature. Protoscoleces thus obtained were washed in Hanks balanced salt solution (HBSS) and were maintained in a sterile preservative solution RPMI-1640. This preservative medium was not amended by any antibiotic or antifungal drugs. Viability/vitality of the protoscoleces was assessed using the trypan blue exclusion technique, prior to any experiments. A 0.01 mL solution of pooled protoscoleces was transferred over a cavity slide and mixed with 0.01 mL of 0.1% aqueous eosin red stain and was evaluated by low power microscopy after 5 min incubation at 37°C. Unstained protoscoleces were considered as viable, while stained protoscoleces were considered as nonviable [29]. When the percentage of viable protoscoleces in the sediment was 95% or more, they were considered to be appropriate for further experiments. 

### 2.7. Anticestodal Activity

In this study, partially purified extracts of endophytic fungi *Pestalotiopsis *spp. were evaluated for scolicidal activity for 10, 20, and 30 min. Two milliliters of each concentration (low concentration 5 to high concentration 30 mg/mL) were placed in a test tube, and a drop of protoscoleces-rich sediment was added to the tube and mixed gently. The tube was then left at room temperature for 10, 20, and 30 min. The supernatant of the solution was then removed with a pipette avoiding settled protoscoleces. Then 2 mL of 0.1% eosin red stain was added to the remaining settled protoscoleces, mixed gently, and incubated at 37°C for 10 min. After incubation the supernatant was discarded and washed with 0.9% PBS to remove the excess stain. The remaining settled protoscoleces were then placed on a glass cavity slide, covered with a cover glass and examined microscopically (Nikon Eclipse E100 research microscope) for viability. The percentages of dead protoscoleces were determined by counting nonviable protoscoleces. Protoscoleces suspended in 0.9% PBS with no exposure to extract were considered as control group and treated with praziquantel (1 *μ*g/mL) that was considered positive control in each experiment. The experiments were performed in triplicate to ensure the reproducibility.

### 2.8. SEM/TEM Analysis

The treated protoscoleces were subjected to transmission electron microscopy (TEM). For TEM analysis, a drop containing 100 **μ**L of protoscoleces rich suspension was placed on carbon-coated copper grids (300 mesh; 3 mm) (TAAB, England, UK), stained with aqueous phosphotungstic acid, subsequently air dried, and visualized under transmission electron microscope (model: Technai 12 G^2^, FEI, The Netherlands) at an accelerating voltage of 120 kV. For SEM analysis, a drop containing 100 *μ*L of treated protoscoleces was fixed in 2.5% glutaraldehyde for overnight. The fixed protoscoleces were then washed again by 0.1 M sodium cacodylate buffer pH 7.4 at 4°C and left for 3 h. The settled protoscoleces were then dehydrated by sequential dipping into alcohol from 30 to 90% followed by a dip into absolute alcohol. The dehydrated protoscoleces were sputter-coated with gold under sputtering device and observed under a Zeiss EVO-LS-10 scanning electron microscope. The images obtained were processed using imaging software SmartSEM VS10.

### 2.9. Statistical Analysis

The statistical package Minitab was used to test for goodness of fit of the two concentrations of the five selected strains towards scolicidal activity at three different incubation times.

## 3. Results

**Figure 1:**
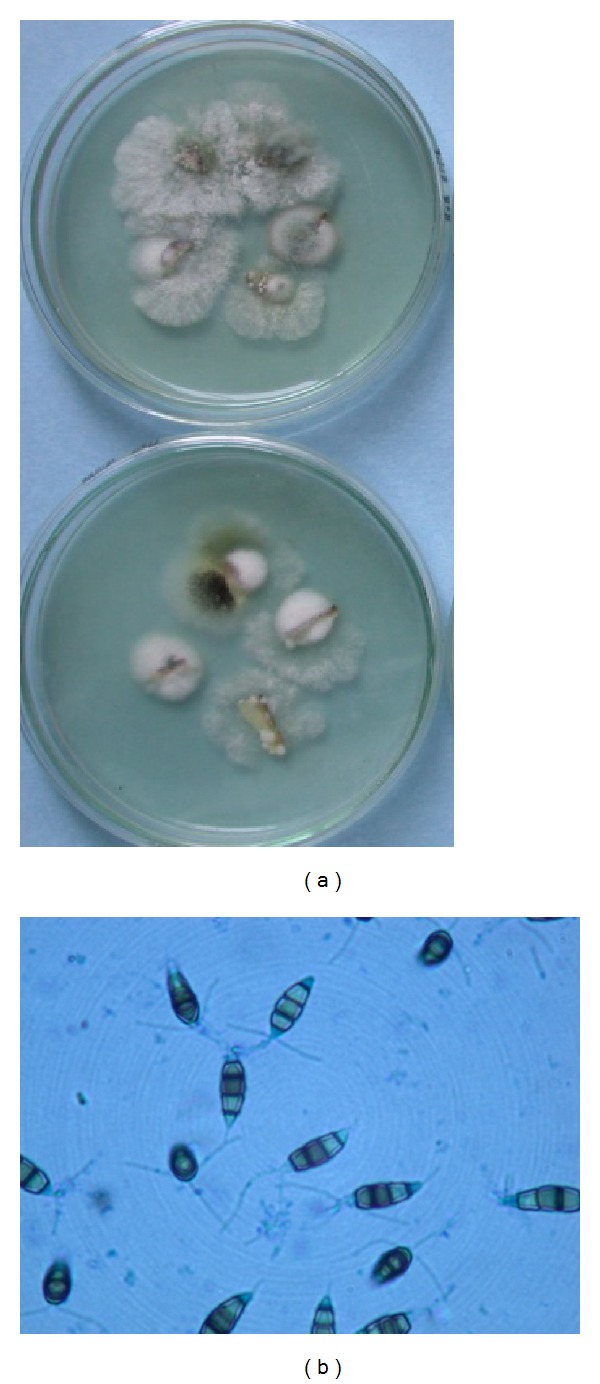
The culture and isolation of endophytic fungi. (a) Petri plates showing emergence of endophytic mycelia from surface-treated tissues of host. (b) The characteristic spores of *Pestalotiopsis* sp. isolated from stem tissues.

**Figure 2:**
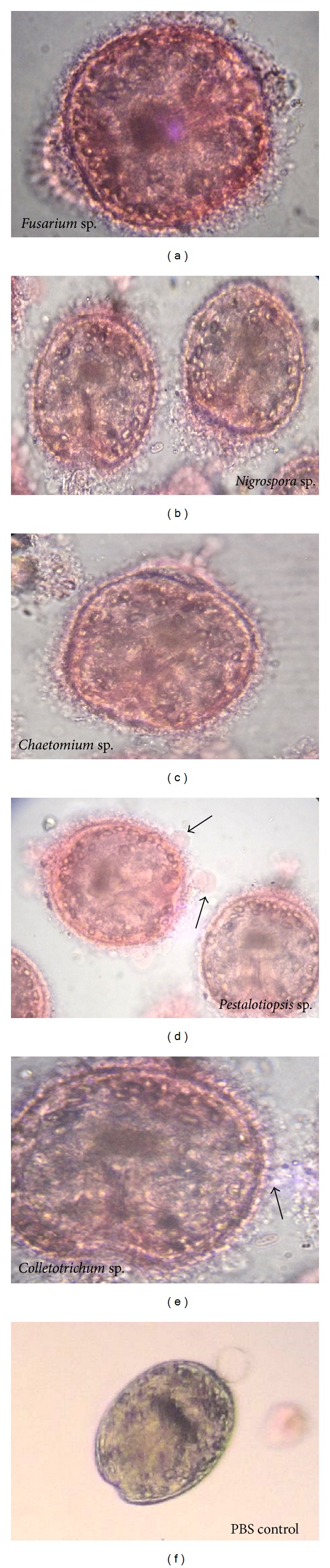
The preliminary screening of selected strains showed marked damage in germinal and tegumental layers and distinct morphological alterations. (d) The digitiform like appendages (arrow) showing considerable tegumental damage leading to viability loss among protoscoleces.

**Figure 3:**
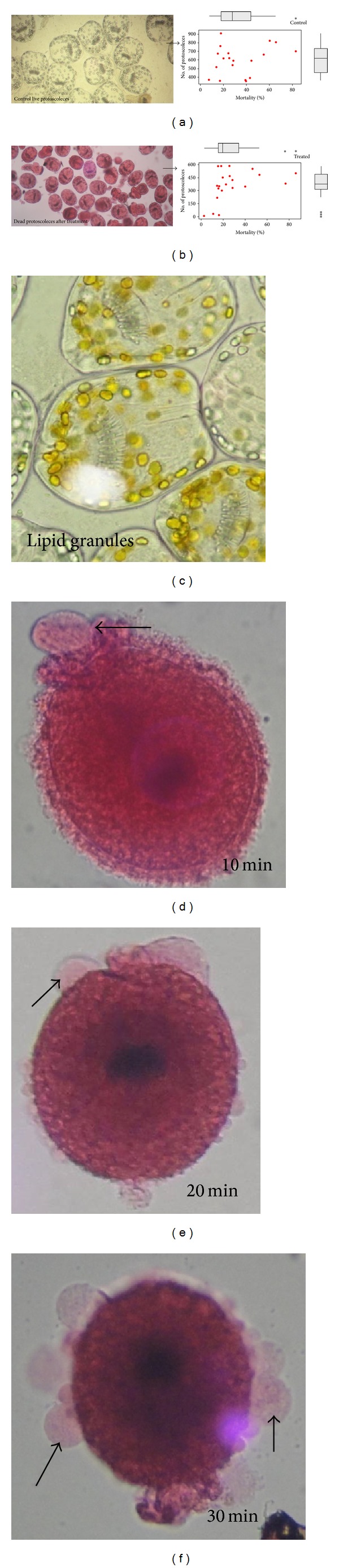
Effect of crude extract of endophytic *Pestalotiopsis* sp. on the viability of protoscoleces of *Echinococcus granulosus. *(a) Control unstained invaginated protoscoleces of hydatid cyst by wet mount drop (10×). (b) Extract-treated (20 mg/mL) hydatid cysts loose their viability and take stain eosin red. ((a)-(b)) represents corresponding mortality observed in control and treated protoscoleces. (c) The lipid granule formation is characteristic feature of the starvation of the protoscoleces. ((d)–(f)) Dead stained protoscoleces after treatment with extract of endophytic *Pestalotiopsis* sp. (f) Extracts showed distinct morphological distortions and degenerative effects and remarkably distinct digitiform tegumental extension after 30 min treatment.

**Figure 4:**
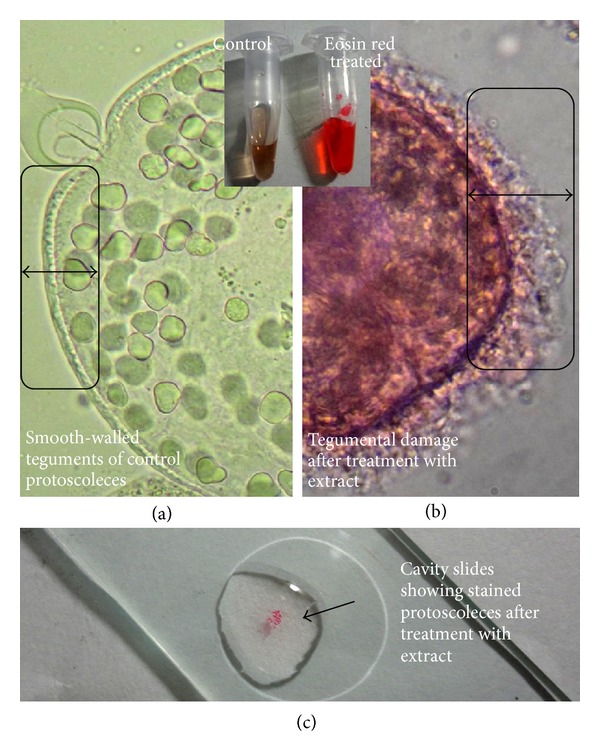
Comparative evaluation of the tegumental and morphological damage in viable and dead protoscoleces after treatment with extract of *Pestalotiopsis* sp. The inset figure represents the treatment with eosin red dye. (a) Control live unstained protoscoleces showing characteristic smooth-walled tegument. (b) *Pestalotiopsis* sp. extract-treated (20 mg/mL; 10 min) protoscoleces showed marked damage in the tegumental layer (notice the inset area). (c) The cavity slide showing the stained protoscoleces preparation for the microscopic examination.

**Figure 5:**
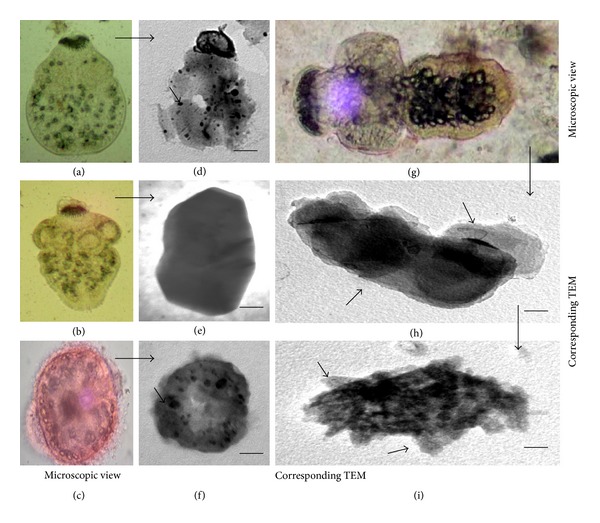
Transmission electron microscopy of extract-treated protoscoleces. (a) Invaginated and (b) evaginated protoscoleces under light microscope. ((d)-(e)) represent corresponding TEM images of protoscoleces, showing complete tegumental damage by marked vacuolization of tegument layer. The distinct digitiform tegumental extensions ((f), (h)-(i)) were also observed under electron microscopy of extract-treated protoscoleces (marked with arrows), (g) corresponding well with normal microscopic view.

**Figure 6:**
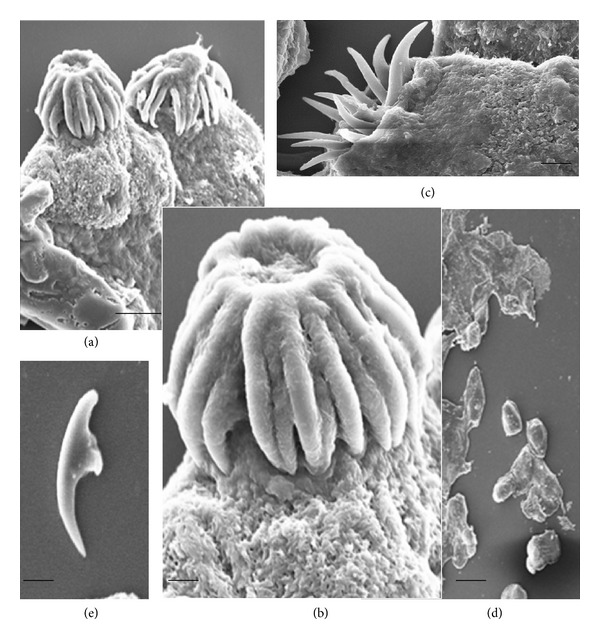
The ultrastructural damages observed with scanning electron microscopy when treated with extract of endophytic *Pestalotiopsis *sp. (a) The rosteller cone disorganization is prominently visible with remarkable damage in scolex and sucker region. (b) The damage at the surface teguments is also observed; loosening of hooks and microtriches was also observed at rosteller cone (c), total disorganization of hooks, and collapse of soma region. (d) The bursting of the soma region indicates the loss of tegumental integrity leading to osmoregulatory damage. (e) Enlarge view of hook, a typical shape characteristic for *E. granulosus*.

This is a first thorough report of anticestodal potential of an endophytic fungus of “Neem” plant against hydatid cysts *Echinococcus granulosus*. We have isolated battery of endophytic microbes from Neem *Azadirachta indica* A. Juss. plants growing in several of its natural habitats, representing both fungal and actinobacterial strains [7, 24]. Several strains were isolated from surface-sterilized stem tissues of the Neem plant; the tissue platted on a PDA plate after incubation up to two weeks, showed emergence of several endophytic hyphae (Figure 1(a)), which were further purified on separate fresh PDA plates. One among them was identified as *Pestalotiopsis* sp. based on its characteristic morphological features. As cottony white colonies show concentric growth, the conidiophores also known as annellide were produced within very compact fruiting bodies representing acervuli of pycnidia. The dark black oozing of the spores at the tip is a characteristic feature of its fruiting bodies. The spores or conidia are 4-5 celled, and the central 2-3 cells were dark brown and with three apical and one distal appendage. These features were among most diagnostic morphological features (Figure 1(b)). In the present study five randomly selected fungal endophytes were chosen to evaluate their anticestodal potential. The extracts (20 mg/mL) of all selected strains were tested against protoscoleces of *E. granulosus* for up to 30 min incubation, and thereafter viability was confirmed with eosin red exclusion test (Table 1). An average scolicidal activity was observed with strains of *Nigrospora *(479 ± 52.9), *Colletotrichum *(469 ± 25.8), *Fusarium *(355 ± 14.5), and *Chaetomium *(332 ± 28.3) showing from 64 to 70% protoscolicidal activity, except *Pestalotiopsis* sp. (581 ± 15.0) which showed promising activity up to 97% mortality just within 30 min of incubation (Table 1). So, this isolate was selected for further study and reestablishment of its potential against hydatid cyst *E. granulosus*. Significant reduction in viability of protoscoleces was observed with all tested strains, the remarkable tegumental damage associated with distinct morphological alterations was also observed (Figures 2(a), 2(b), 2(c), 2(d), and 2(e)), as compared to the PBS control protoscoleces (Figure 2(f)). The extract of endophytic *Pestalotiopsis* sp. represents distinguishing structures as balloon like (digitiform) projections appeared from the altering tegumental layers leading to loss of viability of protoscoleces (Figure 2(d)), while none among the other strains displays his type of activity; however, they also show remarkable tegumental damage. It is quite interesting since this is suggesting that the extract of endophytic *Pestalotiopsis* sp. may contain some metabolite that might be selectively active on tegumental layers of protoscoleces. So, individual experiments with partially purified extract of *Pestalotiopsis* sp. were performed against protoscoleces of *E. granulosus*. The results of *in vitro* vitality and viability tests were also in close accordance with the tissue damage observed at ultrastructural level.  Figure 3 demonstrates the extent of morphological alterations and disintegration of the protoscoleces when treated with 20 mg/mL of *Pestalotiopsis* sp. extract for 30 min. The degenerative changes like rosteller disorganization, loss of hooks, and shedding of microtriches of the scolex region were observed. In addition to these changes in some protoscoleces, the presence of digitiform tegumental extensions appeared on the tegument of the soma region (Figures 3(d), 3(e), and 3(f)). These extensions after further incubation were ruptured and cause severe morphological alterations leading to the loss in viability. There is possibility that these extensions enlarges with due course of time and burst out leading to the disturbance in osmoregulatory system of protoscoleces, that causes loss in viability. Interestingly, as compared to control (Figure 3(a)), 97% mortality was observed (Figure 3(b)) with eosin exclusion test (Figures 3(a) and 3(b)). All protoscoleces died and took eosin red stain, after maximum of 3 h of incubation, without addition of fresh extract during incubation. Resuspension of extract-treated protoscoleces in fresh medium for a period of one week without the extract did not result in any changes, indicating that none of the parasites had survived the treatment. These results demonstrated the dose-dependent protoscolicidal effect of partially purified extract of *Pestalotiopsis* sp. on *E. granulosus*. The primary site of damage observed was the tegument of the parasite. With the increase in the incubation period, the severity of the tegumental damages increase in form of digitiform tegumental extension and presence of numerous blebs in the tegument of the soma region (Figures 3(d)–3(f)). These specific structures can be observed more profusely after 30 min of incubation (Figure 3(f)), together with large number of lipid droplets (Figure 3(c)). Complete loss of morphology was evident due to bursting of extensions, rosteller disorganization and shedding of microtriches. As compared with control teguments (Figure 4(a)), the teguments of treated protoscoleces were severely affected resulting in loss of integrity and ultrastructural alterations such as presence of several blebs on tegument surface (Figure 4(b)). The extract-treated protoscoleces showed positive eosin exclusion test as it takes eosin dye and clearly visible with naked eye in even cavity slides (Figure 4(c)). TEM analysis of the extract treated protoscoleces revealed exact correlation with those of normal microscopic study and exhibit fine details of the ultrastructural changes due to the treatment (Figure 5). The *in vitro* treatment of invaginated protoscoleces caused severe damage markedly observed under SEM/TEM as altered sucker region contracted soma region, ruptured extension leading to burst of protoscoleces (Figure 5(d)) with reference to normal corresponding protoscoleces in microscopic view (Figure 5(a)). Similarly, ultrastructural deformities were also observed with evaginated protoscoleces (Figure 5(e)) and in corresponding microscopic view (Figure 5(b)). The remarkably distinct digitiform tegumental extension was also observed with TEM analysis (Figures 5(h) and 5(i)). The digitiform projections were clearly visible from deforming tegumental layers, very similar as it was observed with normal microscopic view (Figures 5(c), and 5(h)). The combination of all these types of ultrastructural deformities resulted in loss of integrity of the germinal layer, and its alterations contribute to the loss of cyst viability, confirming efficacy of the endophytic *Pestalotiopsis* sp. extract against hydatid cysts. The ultrastructural damage was further confirmed with SEM analysis. The rosteller cone disorganization is prominently visible with remarkable damage in scolex and sucker region; the damage at the surface teguments is also observed, loosening of hooks and microtriches was also observed at rosteller cone (Figures 6(a) and 6(b)), total disorganization of hooks, and collapse of soma region (Figure 6(c)). The bursting of the soma region indicates loss of tegumental integrity leading to osmoregulatory damage and an enlarged view of hook, a typical shape characteristic for *E. granulosus *(Figures 6(d) and 6(e)).

## 4. Discussion

This work describes for the first time the protoscolicidal activity of an endophytic fungus *Pestalotiopsis *sp. isolated from *Azadirachta indica* A. Juss. plant. After establishment of this fact that endophytic *Pestalotiopsis* sp. has significant scolicidal activity by performing screening experiments, we thoroughly studied the partially purified *Pestalotiopsis* extract in further experiments. The unique ultrastructural deformities observed using TEM enabled us to examine the effects induced by the extract of endophytic *Pestalotiopsis* sp. when treated at two different concentrations and incubation period. It is important to note that the associated morphological and ultrastructural changes induced by the extract of *Pestalotiopsis* sp. such as contraction in soma region, formation of blebs on the tegument, rosteller disorganization, loss of hooks, and destruction of microtriches have also been observed with several established protoscolicidal compounds including praziquantel [30–32], benzimidazole [31, 33], and ivermectin [34]. Rosteller disorganization and destruction of microtriches might be major reason associated with loss of viability of protoscoleces as they are directly interface with the establishment of parasite for nutrient absorption [29] and defense. Similarly, the digitiform tegumental extensions were also observed in protoscoleces treated with praziquantel under similar experimental conditions; this indicates that the bioactive fraction in our crude extract might have some compound that have activity like pyrazinoisoquinoline derivative, but it need further chemical characterization through bioassay-guided fractionation. However, in some recent studies, it was observed that praziquantel have substantial effect when used in combination of other scolicidal agents such as albendazole [35]. We may also speculate about increased efficacy of our extract from endophytic *Pestalotiopsis* sp. when used in combination of praziquantel and/or albendazole as this may induce similar type of ultrastructural changes with praziquantel [36]. Increasing vacuolization, small lamellated bodies, and presence of lipid droplets in treated protoscoleces indicate general tissue stress leading to a stage prior to necrosis. Thus, the presence of lipid droplets in the cytoplasm of tegumentary cells of the germinal layers indicates metabolic disruption of the cyst [34, 37, 38], and very similar tissue degeneration was observed, when treated with netobimin [39] and isoprinosine [40]. What is unique with this study is that it is a first exhaustive attempt to screen endophytic fungi for parasiticidal activity against *E. granulosus*. Endophytes have recently been recognized as an unexplored biofactory of functional metabolites [3, 5] that have array of activities against existing and emerging new threats of biomedical concern. But unfortunately, very few efforts have been done for finding novel metabolites against helminthes parasite from endophytic fungi. The present study is an attempt to screen out novel bioactive leads to be developed as new prodrug for helminthes parasites. The initial results obtained is quite encouraging since the extract of endophytic *Pestalotiopsis* sp. shows activity very similar to some existing scolicidal drugs and can be further studied for its complete chemical characterization leading to the identification of the molecule concern for this activity. We are currently working with the crude extract for purification and characterization of bioactive fractions of endophytic *Pestalotiopsis* sp. and expecting some novel chemistry that exerts these activities in its purified form. Additionally, more exhaustive evaluation of our fungal extract chemotherapeutic efficiency will be investigated *in vivo* conditions using animal models in future study.

## Figures and Tables

**Table 1:** Preliminary screening results of the selected endophytic strains against hydatid cyst *Echinococcus granulosus. *Endophytic *Pestalotiopsis* sp. extract shows promising activity.

Endophytic microbes (crude extract 20 mg/mL)	Protoscolicidal activity (mortality^a^) after incubation up to 30 min								
	10 min			20 min			30 min		
	Control	Dead	M**	Control	Dead	M**	Control	Dead	M**
*Nigrospora *sp.	623 ± 25.1	348 ± 40.1	58	825 ± 60.1	500 ± 86.0	62	706 ± 82.7	479 ± 52.9	70
*Pestalotiopsis *sp.	366 ± 39.0	299 ± 18.8	85	357 ± 16.8	323 ± 15.5	**94**	621 ± 20.0	581 ± 15.0	**97**
*Colletotrichum* sp.	372 ± 7.37	216 ± 14.5	59	674 ± 15.0	425 ± 28.3	66	677 ± 24.4	469 ± 25.8	70
*Fusarium *sp.	762 ± 17.5	455 ± 19.5	61	913 ± 17.7	586 ± 25.6	66	541 ± 27.7	355 ± 14.5	69
*Chaetomium *sp.	663 ± 54.9	382 ± 76.6	59	917 ± 17.7	550 ± 46.7	63	552 ± 26.7	332 ± 28.3	64
Praziquantel	391 ± 43.1	376 ± 22.5	97	354 ± 39.5	348 ± 16.2	99	592 ± 44.2	584 ± 18.1	100
PBS*	591 ± 28.5	8 ± 2.60	*∼*1	812 ± 65.3	29 ± 11.0	*∼*2	517 ± 13.8	15 ± 16.1	*∼*4

*PBS: phosphate buffer saline; **M: percentage mortality; ^a^as determined by adopting eosin red exclusion method (see material and methods for detail).
